# Differential mercury accumulation and links to blood mercury levels across feather types in a long-lived seabird

**DOI:** 10.1007/s10661-026-15310-4

**Published:** 2026-04-15

**Authors:** Justine Bertram, Elias Garsi, Coraline Bichet, Nathalie Kürten, Peter J. Schupp, Sandra Bouwhuis

**Affiliations:** 1 Institute of Avian Research, An Der Vogelwarte 21, 26386 Wilhelmshaven, Germany; 2 Centre d’Etudes Biologiques de Chizé (CEBC), CNRS-La Rochelle Université, Villiers-en-Bois, France; 3 Department for Chemistry and Biology of the Marine Environment, Carl Von Ossietzky Universität Oldenburg, 26382 Wilhelmshaven, Terramare Germany; 4 Helmholtz Institute for Functional Marine Biodiversity at the University of Oldenburg, 26129 Oldenburg, Germany

**Keywords:** Common tern, Bioaccumulation, THg, Plumage, Hg, Biomonitoring

## Abstract

Mercury is a pervasive pollutant that readily enters the food web, accumulates within organisms and across trophic levels, and poses a significant threat to long-lived top predators, such as many seabirds. Since mercury can be deposited in feathers and therefore excreted through moult, feather analysis offers a minimally invasive way to assess mercury contamination levels in birds. However, it remains largely unclear whether mercury levels vary among feather types within individuals, how feather mercury levels relate to those in blood, and whether all levels vary with individual characteristics, such as sex and age, in a similar way. To address these issues, we collected dorsal, ventral, primary and tail feathers from > 200 common terns (*Sterna hirundo*) from a breeding population in the north of Germany, of which a subset was blood sampled as well. Additionally, we collected multiple dorsal feather samples from 313 individuals across years (i.e. longitudinally), to assess whether age effects were likely to reflect within-individual processes. Mercury levels were significantly correlated across feather types within individuals, with those in dorsal and ventral feathers, both grown in the wintering area, also correlating with mercury levels in blood sampled during the breeding season. Males and females did not differ in their feather mercury levels, but older birds exhibited higher mercury levels in all feather types except for tail feathers. Unique analysis of the longitudinally collected dorsal feathers showed that although the within-individual estimate for age was not significant itself, it was very similar to the among-individual age effect, suggesting that within-individual accumulation of mercury during ageing is a likely explanation for older birds having higher mercury levels in most of their feathers. We suggest that body feathers are most suitable for characterising the mercury levels of seabirds in a little-invasive way, but that being able to account for the age of the sampled individuals is important.

**Supplementary Information:**

The online version contains supplementary material available at 10.1007/s10661-026-15310-4.

## Introduction

Mercury is a global pollutant that readily enters organisms through the diet and can adversely affect health and reproduction (Whitney & Cristol, 2017). Because mercury both accumulates within individuals over time (Bertram et al., 2024a) and biomagnifies across trophic levels (Atwell et al., 1998), long-lived top predators, such as many seabirds, face particularly high exposure (Chételat et al., 2020; Mills et al., 2022), making them a priority for biomonitoring efforts (Burger & Gochfeld, 2004). Such biomonitoring of live seabirds can be done not only by collecting blood samples (e.g.Cruz-Flores et al., 2024; Lemesle et al., 2024; Schnelle et al., 2025) but also by using their eggs (Ackerman et al., 2020; Bond & Diamond, 2009), which reflect the maternal mercury burden at the time of egg laying (Ackerman et al., 2016; Beccardi et al., 2025; Bertram et al., 2025a). Feathers represent yet another option (Monteiro & Furness, 1995; Sun et al., 2022), as they integrate mercury ingested during their growth (Bearhop et al., 2000; Bottini et al., 2021), as well as mercury remobilized from body stores since the previous moult (Braune & Gaskin, 1987; Furness et al., 1986).

Feathers are particularly advantageous for biomonitoring because they remain metabolically inert once formed, are easy to store, and can be collected little-invasively (Appelquist et al., 1984). On the other hand, however, feather mercury concentrations can be variable, both among (Carravieri et al., 2014a; Padilha et al., 2023; Polito et al., 2016; Pollet et al., 2022; Quillfeldt et al., 2023; Souza et al., 2020) and within species (Bond & Diamond, 2008; Furness et al., 1986; Gochfeld, 1980; Pedro et al., 2015). Likewise, mercury concentrations can also differ among feather types within individuals (Furness et al., 1986; Braune & Gaskin, 1987; Eagles-Smith et al., 2008; Misztal‐Szkudlińska et al., 2012; Carravieri et al., 2014b; Bighetti et al., 2022; Canova et al., 2025), and even among individual feathers within a single feather type (Bertram et al., 2022; Bond & Diamond, 2008). Part of this variability is likely linked to the sequence of feather formation (Bighetti et al., 2022). Feathers grown at different times reflect distinct mercury pools: those formed early in the moult cycle primarily integrate mercury accumulated since the previous moult and remobilized from internal tissues (Braune & Gaskin, 1987; Furness et al., 1986; Renedo et al., 2021), whereas those grown later likely are more strongly influenced by mercury ingested during feather growth (Bearhop et al., 2000; Bottini et al., 2021). Additionally, different feathers grow at different rates (Jenni et al., 2020), such that those that develop more slowly might integrate mercury obtained from a larger range of foraging areas and dietary sources if birds remain mobile during moult or food availability is temporally variable (Gatt et al., 2021; Peterson et al., 2019).

Whereas moult sequence effects are expected to be general, individual characteristics of the sampled birds may additionally influence feather mercury levels. Age and sex are prime candidates in this respect, given that these have been found to affect blood mercury levels (Bertram et al., 2024a), which themselves have been shown to correlate with those in feathers (Ackerman et al., 2012; Bearhop et al., 2000). Some studies indeed have reported higher mercury levels in adult feathers compared to those of chicks (Burger & Gochfeld, 2000; Burger et al., 2001; Carravieri et al., 2013, 2014a; Polito et al., 2016; Souza et al., 2020; Bighetti et al., 2021, 2022; Dodino et al., 2022; Canova et al., 2025). However, chicks and adults differ not only in age but also in growth, in diet—with chicks often feeding on smaller, less contaminated prey—and in moult patterns, as chick feathers grow simultaneously at the hatching site, while adult feathers are often replaced sequentially and partly in winter. Among adult birds, studies have found either no relationship between age and feather mercury (Burger et al., 1994; Thompson et al., 1993), a positive correlation (Bertram et al., 2024b), or even a negative correlation (Bajracharya et al., 2022; Bustamante et al., 2016; Rewi et al., 2024; Tavares et al., 2013). While sex differences in feather mercury levels appear negligible in several species (Bertram et al., 2022; Bighetti et al., 2021; Canova et al., 2025; Polito et al., 2016; Souza et al., 2020; Thompson et al., 1991), it is poorly studied whether any age effects may be sex-specific, such as in blood (Bertram et al., 2024a), and whether patterns hold across feather types.

We study mercury levels in common terns (*Sterna hirundo*) from a monospecific breeding colony on the North Sea coast of Germany, a long-term study population in which all locally hatched chicks have been ringed and sexed since 1992. Here, we investigate (i) how mercury levels correlate across different feather types, (ii) how feather mercury levels relate to those in blood, and (iii) how mercury levels in the different feather types vary with age and/or differ between males and females. To do so, we collected dorsal, ventral, primary, and tail feathers from 201 individuals of known sex and age that died during outbreaks of highly pathogenic avian influenza in the breeding seasons of 2022 and 2023 (Bouwhuis, 2025; Ewing & Bouwhuis, 2025). For a subset of 89 of these individuals, blood samples were also collected prior to death. In addition, we analysed 510 dorsal feather samples collected from 313 individuals between 2017 and 2023, as part of a larger study on mercury accumulation in adults (Bertram et al., 2022, 2024a, 2024b) and mercury transfer to offspring (Bertram et al., 2025a, 2025b). Using these unique longitudinal data, we decompose the age effect found for this feather type to its within- and among-individual components, allowing us to assess whether it is most likely to be underpinned by within-individual processes, such as age-specific mercury accumulation, or among-individual processes, such as selective (dis)appearance of individuals with either low or high mercury levels. Based on previous work showing long-term repeatability of mercury levels suggestive of consistent foraging strategies or excretion across life (Bertram et al., 2024b), we expected mercury levels to be positively correlated across feather types and with those in blood. We further predicted that feather mercury concentrations would not show strong differences between males and females (Bertram et al., 2022), but would be higher in older birds, reflecting within-individual mercury accumulation previously found to occur in blood (Bertram et al., 2024a).

## Methods

### Study species and site

We investigated mercury contamination of common terns breeding at a monospecific colony situated at the Banter See (53°30′40″ N, 08°06′20″ E) in Wilhelmshaven, Germany. Common terns are long-lived, long-distance migratory seabirds, whose diet mainly consists of fish of a length of up to 15 cm (Becker & Ludwigs, 2004). Birds from the studied population show strong fidelity both to the Banter See breeding colony (Szostek & Becker, 2012) and to their wintering areas, which are scattered along the West- to South-African coast (Kürten et al., 2022). The colony has been monitored intensively since 1992, with all locally hatched chicks being ringed, and all fledglings additionally receiving a subcutaneously implanted transponder that allows for their lifelong automatic detection at the colony using an antenna system (Becker & Wendeln, 1997). Sexing of fledglings follows molecular procedures described by Becker and Wink (2003).

Common terns undergo two sequential moults per year: a complete post-nuptial moult, starting with the first primary (P1) and first rectrix (R1) between July and August, and a partial pre-nuptial moult, replacing body feathers and other types, usually between December and February (Becker & Ludwigs, 2004). We analysed mercury concentrations in feathers grown during both moults: the second primary (P2) and first rectrix (R1), which are likely formed at—or close to—the breeding area (Walters, 1979), as well as dorsal and ventral body feathers, which are likely formed in the wintering areas (Dwight, 1901).

### Data collection

We collected dorsal, ventral, tail, and primary feathers from 201 locally hatched and recruited adult common terns that thus were of known identity, sex, and age and that died of avian influenza during the breeding seasons of 2022 and 2023. Birds had been stored frozen for approximately two years prior to analysis and were processed while still largely frozen during feather sampling. Disposable gloves and an FFP2 mask were worn throughout sampling and subsequent sample handling until completion of the washing procedure. For a subset of 89 of these birds, blood was sampled prior to death, during incubation, using Mexican kissing bugs (*Dipetalogaster maxima*; Becker et al., 2006). One or two starved instars were inserted into a hollow artificial egg that was placed in the nest of a focal bird, and the bugs were allowed to feed for approximately 20–30 min through small holes along the surface of this egg. After feeding, the bugs’ blood meals were extracted with a syringe and stored at −18 °C until analysis. To confirm that this method did not influence mercury concentrations measured in the blood (see below), we sampled blood from the brachial vein of 18 birds that previously have been ‘bug-sampled’ as well. Comparing the mercury measurements from the blood samples obtained in both ways yielded a correlation of 93% (Bertram et al., 2024a).

As part of a tracking project, breeding birds were caught between 2017 and 2022 using electronically released drop traps (see Kürten et al., 2022). During handling, five to ten dorsal feathers were collected from each individual (Bertram et al., 2022, 2024b), resulting in 309 samples from 148 live birds, several of which were sampled repeatedly across years. Of the 201 birds sampled post-mortem (see above), 165 individuals had not been sampled previously, whereas 36 individuals also occurred within the ‘live-bird dataset’, providing both longitudinal samples from earlier years and a final sample collected after death. As such, this second dataset comprised a total of 510 longitudinally collected dorsal feather samples of 313 individuals. Given that the outbreaks of avian influenza took place during the breeding season, whereas the dorsal feathers we collected were grown at the wintering grounds, we did not necessarily expect the mercury levels to differ between feathers collected from live or dead birds. This indeed was the case: the mercury levels for the dorsal feathers collected from the 36 birds sampled both before (3.33 ± 0.18 μg·g^−1^ dw) and after (3.52 ± 0.22 μg·g^−1^ dw) death did not differ significantly from one another.

### Mercury analysis

Total mercury (THg) concentrations were measured using a Direct Mercury Analyzer (DMA-80, MLS Mikrowellen-Labor-Systeme GmbH, Germany). Feathers were cleaned to remove potential external contamination using three alternating rinses in distilled water and acetone, followed by a 15-min ultrasonic cleaning in each solution using a Sonorex Super RK510H bath (Bandelin, Germany). Blood samples required no preparation.

For analysis, we weighed either 1–2 body feathers (dorsal or ventral) or, for primary and tail feathers, the distal third of a single feather, and wrapped them in mercury-free aluminium foil. The foil-wrapped feather samples, or c. 20 μl of whole blood, were then inserted into nickel sample boats and thermally decomposed at 750 °C in the DMA-80. During this process, the released gaseous mercury was trapped on a gold amalgamator, while the other sample components were combusted. The amalgamator was then heated to 850 °C to release the mercury, and quantification was carried out by atomic absorption spectrometry at 253.7 nm. While mercury concentrations in feathers were determined in dry weight (dw), those in blood were determined in wet weight (ww).

Each analytical run included a blank to confirm the absence of instrument contamination, as well as a certified reference material (DORM-3 or DORM-4; fish protein from the National Research Council of Canada, with certified mercury concentrations of 382 or 412 μg⋅kg^−1^ dry weight). Mean recoveries (± SD) were 103 ± 9% and 116 ± 6%, respectively, and measured mercury concentrations were corrected when recoveries deviated from the certified values. All dorsal feather samples were analysed in triplicate, and 25 samples of each other feather type in duplicate to ensure measurement reliability. For primary and tail feathers, the proximal part was used for these replicate measurements. The intraclass correlation for dorsal feathers was ICC = 73.5% (CI 70.2–76.7), and correlations between duplicate measurements were strong for ventral (R = 84.1%, CI 66.9–92.8), primary (*R* = 95.0%, CI 88.8–97.8), and tail feathers (R = 74.6%, CI 49.7–88.1) as well (Figure S1). For statistical analyses, only the first THg measurement per feather type and individual was used.

### Statistical analyses

Statistical analyses were performed, and figures produced, in R (version 2025.5.0.496, R Core Team, 2025). Linear (mixed) models were fitted using the package *lme4* (Bates et al., 2015), and significance testing of fixed effects was carried out with *lmerTest* (Kuznetsova et al., 2017), with *p* < 0.05 considered indicative of statistical significance. Figures were drawn with *ggplot2* (Wickham, 2016). THg levels were log-transformed to meet model assumptions, which we confirmed visually using histograms.

To examine correlations among THg levels in dorsal, ventral, primary, and tail feathers of the 201 birds that were sampled after they had died from avian influenza, as well as with blood THg levels obtained for 89 of these birds, we first calculated pairwise Pearson correlations and visualized the resulting correlation matrix using the *ggpairs* function (Schloerke et al., 2025). To investigate potential (sex-specific) correlations among feather THg levels and age, we ran four linear models, one for each feather type, with feather mercury concentration as the dependent variable. Based on results from previous analyses of dorsal feathers only (Bertram et al., 2024b), the initial model included linear age (‘age’) and quadratic age (‘age^2^’) as covariates. In addition, because feather THg levels have been shown to vary significantly among years (Bertram et al., 2022), ‘year’ was included as a fixed effect, together with ‘sex’ and the interactions between ‘sex’ and both age terms. Non-significant interactions were removed through stepwise backwards elimination, but main effects were kept because we wanted to specifically assess their parameter estimates. In addition, we kept the non-significant effect for ‘age^2^’ for the tail feathers (see Results), because this effect was significant for the other feather types, and its inclusion ensured structural comparability among models.

For the dorsal feathers making up 510 samples, which were collected longitudinally across seven years for 313 birds, we additionally applied a linear mixed model with ‘individual identity’ and ‘year’ as random effects to account for repeated measurements and annual variation, respectively, and ‘sex’ as a fixed effect. This model allowed us to separate and compare variation resulting from differences among individuals from changes occurring within individuals as they aged. Hereto, we applied the within-subject centring approach described by van de Pol and Wright (2009) and split each bird’s age into its ‘average age’ across all sampling occasions and its ‘delta age’, defined as ‘actual age’–‘average age’. When doing so, ‘average age’ represents the among-individual (population-level) relationship between age and THg levels, whereas ‘delta age’ captures how THg levels change within an individual as it grows older. The initial model additionally included the interaction between both age components to test for non-linear within-individual effects of age (i.e. accelerating or decelerating bioaccumulation), as well as the interaction between ‘delta age’ and ‘sex’ to test whether within-individual accumulation patterns differed between males and females. We then again applied the approach of stepwise backward elimination to obtain a minimal adequate model.

## Results

Total mercury (THg) concentrations measured in a total of 1113 feather samples collected from 313 individuals ranged from 0.17 to 35.15 μg⋅g^−1^, with ventral feathers showing the highest mean THg levels (3.80 ± 0.13 μg·g^−1^ dw) and tail feathers the lowest (2.50 ± 0.14 μg·g^−1^ dw; Table 1). Across all feather types, approximately 18% of samples showed mercury levels that fell within the no-risk category, 65% levels that fell within the low-risk, 16% levels that fell within the moderate-risk, and fewer than 1% levels that fell within the high- or severe-risk categories for health effects according to Chastel et al. (2022) (Table 1).

**Table 1:** Sample details (collection years and sample size, mean total mercury (THg) concentration ± standard errors in μg⋅g^−1^ dw, as well as minima and maxima), together with the distribution of samples across toxicity risk categories provided by Chastel et al., (2022; no risk: < 1.62 μg·g^−1^, low risk: 1.62–4.53 μg·g^−1^, moderate risk: 4.53–9.14 μg·g^−1^, high risk: 9.14–10.99 μg·g^−1^, and severe risk: > 10.99 μg·g^−1^ THg), for dorsal, ventral, primary, and tail feathers of common terns. Note that dorsal feathers were collected from 201 birds that died of avian influenza and were sampled for the other feather types as well (first line), as well as from 148 individuals that were healthily incubating their clutches during the long-term study period (309 additional samples)

Feather type	Collection years (*n*)	Mean THg ± SE	Min THg	Max THg	Number of samples within risk categories				
					None	Low	Moderate	High	Severe
Dorsal	2022–2023 (201)	3.60 ± 0.15	0.87	21.32	13	139	47	1	1
	2017–2023 (510)	3.47 ± 0.09	0.77	35.15	51	341	115	2	1
Ventral	2022–2023 (201)	3.80 ± 0.13	1.25	18.79	22	148	30	0	1
Primary	2022–2023 (201)	3.43 ± 0.16	0.69	23.91	46	126	28	0	1
Tail	2022–2023 (201)	2.50 ± 0.14	0.22	19.05	83	110	6	0	2

**Fig. 1:**
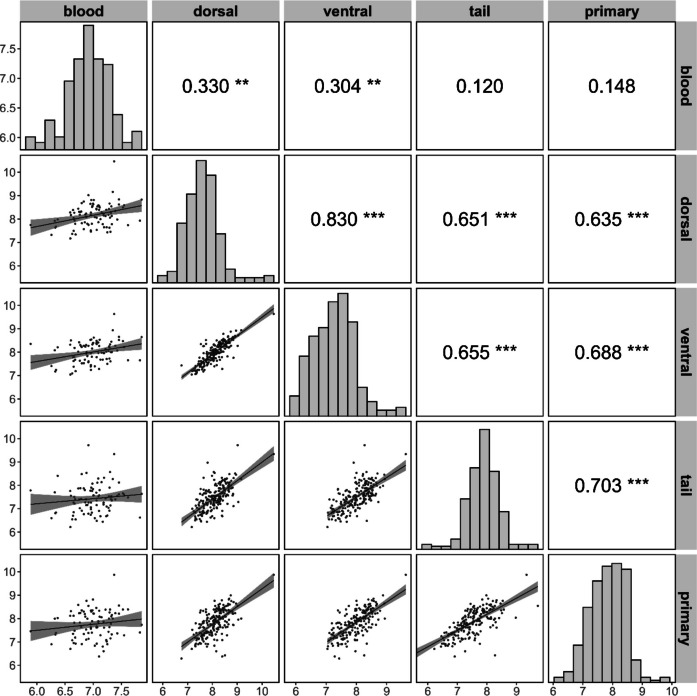
Pairwise correlations between log-transformed total mercury (THg) concentrations in blood and four feather types (dorsal, ventral, tail, and primary) of common terns. Lower panels show scatterplots with fitted linear regression lines and 95% confidence intervals, while diagonal panels display the data distributions, and upper panels provide Pearson’s correlation coefficients with significance levels indicated by asterisks (*p < 0.05, **p < 0.01, and ***p < 0.001)

**Fig. 2:**
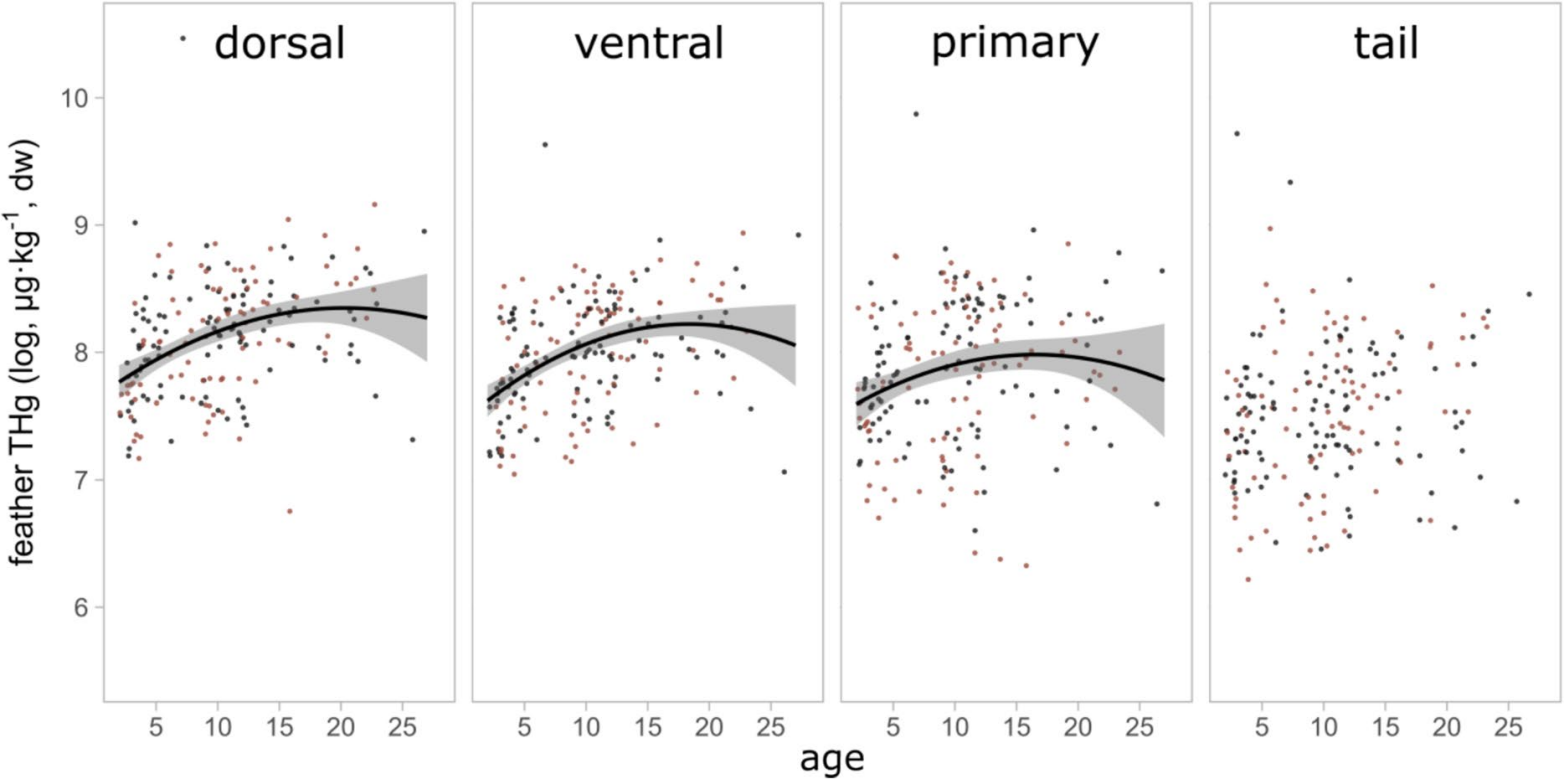
Total mercury (THg) concentrations of feathers of 201 male (black) and female (red) common terns in relation to their age (in years). Points represent the raw data, lines the model predictions for significant correlations, and shaded areas the 95% confidence intervals around the predicted relationships

Within individuals, THg concentrations among the different feather types were significantly positively correlated, and THg levels in both dorsal and ventral, but not tail or primary, feathers showed significant correlations with those in blood as well (Fig. 1). THg concentrations did not differ between males and females, but older birds exhibited significantly higher THg concentrations in their dorsal, ventral, and primary, but not tail, feathers compared to younger individuals (Table 2, Fig. 2).

**Table 2:** Estimates from models testing whether total mercury levels (THg) of dorsal, ventral, primary, or tail feathers (each *n* = 201) differ between the sexes or correlate with age in common terns. Reported results are obtained from the model that retained the main effects of ‘year’, ‘sex’, ‘age’, and ‘age^2^’ independent of their significance. Point estimates of fixed effects are shown as posterior means and [95% credible intervals]; *p*-values for significant effects (*p* < 0.05) are printed in bold

	Dorsal feathers		Ventral feathers		Primary feathers		Tail feathers	
Fixed effects	Estimate [95% CI]	*p*-value	Estimate [95% CI]	*p*-value	Estimate [95% CI]	*p*-value	Estimate [95% CI]	*p*-value
Intercept	7.648 [7.426, 7.852]	** < 0.001**	7.484 [7.290, 7.672]	** < 0.001**	7.629 [7.360, 7.904]	** < 0.001**	7.538 [7.255, 7.803]	** < 0.001**
Year	−0.036 [−0.167, 0.115]	0.618	−0.051 [−0.180, 0.073]	0.444	−0.283 [−0.462, −0.108]	**0.002**	−0.291 [−0.488, 0.095]	**0.002**
Sex	−0.012 [−0.137, 0.108]	0.843	0.008 [−0.102, 0.1117]	0.892	−0.119 [−0.265, 0.030]	0.124	−0.042 [−0.192, 0.114]	0.617
Age	0.070 [0.031, 0.111]	** < 0.001**	0.081 [0.047, 0.114]	** < 0.001**	0.060 [0.014, 0.110]	**0.013**	0.003 [−0.046, 0.054]	0.892
Age^2^	−0.002 [−0.003, −0.0001]	**0.026**	−0.002 [−0.004, −0.001]	**0.002**	−0.002 [−0.004, −0.00005]	**0.047**	0.0002 [−0.002, 0.002]	0.832

**Fig. 3:**
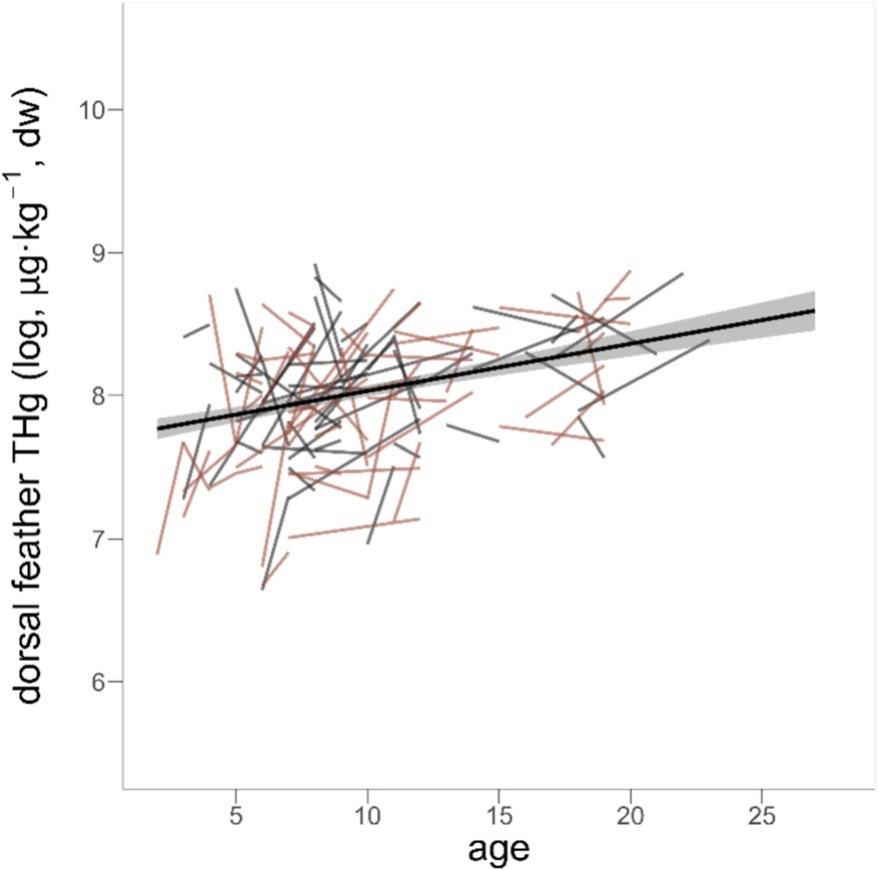
Total mercury concentrations in 510 dorsal feather samples of 313 male (black) and female (red) common terns in relation to age (in years). Thin lines connect repeated measurements within individuals across sampling events (represented by the ‘delta age’ term in the models), the thick line the significant average age effect, and the shaded area the 95% confidence interval around the predicted relationship

Using the longitudinally collected dorsal feathers, we found a correlation between THg and the among-individual component of average age (Table 3). The estimate for the within-individual delta age term was nearly identical to that of the among-individual average age term, but did not reach statistical significance (Table 3, Fig. 3). Given that it is well known that the power to detect a delta age effect is lower than that to detect an average age effect because of the reduced age range to do so (Nussey et al., 2008), we interpret the similarity of the estimates to suggest that the overall among-individual pattern of older birds having higher levels of feather mercury may well be explained by the within-individual process of bioaccumulation.

**Table 3:** Estimates from the model testing whether within- and/or among-individual age effects shape total mercury (THg) concentrations in 510 samples of dorsal feathers obtained from 313 common terns. Reported results are obtained from the model that retained the main effects of ‘average age’, ‘delta age’, and ‘sex’ independent of their significance. Point estimates of fixed and random effects are shown as posterior means and [95% credible intervals]; *p*-values for significant effects (*p* < 0.05) are printed in bold

Fixed effects	Estimate [95% CI]	*p*-value
Intercept	7.684 [7.527, 7.835]	** < 0.001**
Sex	−0.032 [−0.124, 0.060]	0.531
Average age	0.033 [0.025, 0.042]	** < 0.001**
Delta age	0.038 [−0.008, 0.083]	0.109
**Random effects**		
Individual identity	0.086 [0.073, 0.100]	
Year	0.016 [0.007, 0.030]	
Residual	0.120 [0.107, 0.136]

## Discussion

In this study, we analysed 1113 feather samples from 313 common terns of known sex and age to examine how total mercury levels in dorsal, ventral, primary and tail feathers correlate among each other and with those in blood, and whether they vary with age or differ between males and females in a similar way. Mercury concentrations showed substantial variation. Across all feather types, the majority of samples showed mercury levels below the admitted toxicity threshold or within the low-risk category (Chastel et al., 2022). However, a non-negligible proportion of feathers (c. 17%) showed levels that fell into moderate or higher risk categories, and mean mercury concentrations in body and primary feathers exceeded those reported for other common tern colonies (body feathers: Burger et al., 1994; Bond and Diamond, 2009; Bracey et al., 2020; primary feathers: Braune, 1987). This emphasizes the potential ecological relevance of mercury exposure in this population, both at the breeding site (for primary and tail feathers) and at the wintering grounds (for body feathers). Additionally, we detected significant correlations among mercury levels in all feather types and between those in body feathers and blood. As predicted, we found no difference in feather mercury levels between sexes, whereas age emerged as an important factor explaining variation in mercury levels in body and primary feathers. Longitudinal analyses of dorsal feathers further suggested that the fact that older birds exhibit higher mercury levels than younger birds may be driven by within-individual accumulation as birds grow older, although this is based on the parameter estimates for the among- and within-individual effects of age being nearly identical, whereas the within-individual effect did not reach statistical significance itself.

Mercury concentrations were significantly positively correlated across feathers of all four feather types, with the strongest correlation observed between those in dorsal and ventral feathers, as also found in another seabird species, the brown booby (*Sula leucogaster*; Bighetti et al., 2022). Stronger correlations among THg levels of body (dorsal and ventral) feathers are plausible given their faster growth and therefore lower intra-feather variability, compared to primary and tail feathers, which grow over a longer period of time (Jenni et al., 2020), potentially increasing variability (Gatt et al., 2021; Peterson et al., 2019). Additionally, both dorsal and ventral feathers are produced late in the moult cycle (Becker & Ludwigs, 2004), when much of the previously accumulated mercury might already be excreted. This does not mean that their overall THg levels are lower, but that these levels are more likely to primarily reflect recent levels of dietary intake of mercury in the wintering areas (Bearhop et al., 2000; Bottini et al., 2021) and are therefore more strongly influenced by an individual’s foraging ability and prey preferences at this part of the annual cycle and range. In contrast, primary and tail feathers grow more slowly and shortly after the breeding season, thereby potentially integrating mostly the mercury accumulated since the previous moult (Braune & Gaskin, 1987; Furness et al., 1986; Gatt et al., 2021), but also being subject to more within-individual variation if foraging areas or prey availability shows increased variability during this time.

Mercury levels in dorsal and ventral, but not primary or tail, feathers correlated with those in blood. This too may be explained by pollution levels in body feathers, as well as blood (e.g. Bracey et al., 2020; Gilmour et al., 2019), but not in primary and tail feathers (see above), reflecting short-term dietary mercury exposure during feather growth (Bearhop et al., 2000; Bottini et al., 2021). Mercury levels in body feathers may therefore indicate an individual’s (repeatable) foraging ecology: high values could occur in particularly efficient foragers that exploit more nutritious but also more contaminated fish, both in the wintering areas, where the body feathers are grown, and, in the breeding area, where the blood sample is taken, and/or reflect consistent prey preferences, with individuals feeding on larger or higher-trophic-level prey species tending to show higher mercury concentrations in both blood and body feathers (Bertram et al., 2024b). Analysing stable isotopes in both tissues, and linking those to mercury levels could help test this hypothesis. In contrast, mercury concentrations in primary and tail feathers appear less influenced by blood mercury at the time of feather growth and may instead be shaped by internal redistribution from storage tissues. Linking mercury in these feathers to mercury concentrations in storage tissues, such as muscle, liver or kidneys (Thompson et al., 1991; Eagles-Smith et al., 2008; Renedo et al., 2021), could provide insights into whether this indeed is the case.

Although we did not detect any sex effects, coherent with findings from previous studies (Bertram et al., 2022; Bighetti et al., 2021; Canova et al., 2025; Polito et al., 2016; Souza et al., 2020; Thompson et al., 1991), older birds exhibited significantly higher mercury levels in their body and primary feathers compared to younger individuals. This aligns with our previous finding of blood mercury also being higher in older birds, due to within-individual accumulation as birds grow older (Bertram et al., 2024a). For the body feathers it may even be a direct effect of the blood-feather correlation among THg levels, whereas in primary feathers, it may indicate that older birds have higher mercury levels not only in their blood but also in their internal tissues, from which mercury is likely remobilized and excreted into primary feathers. The absence of a comparable age effect in tail feathers, however, is puzzling, given that primary and tail feathers grow roughly simultaneously and would be expected to incorporate mercury from similar sources.

To further explore the mechanisms underlying the age-related pollution pattern in body feathers, we analysed THg levels in longitudinally collected dorsal feathers. Although the estimate for the within-individual process of bioaccumulation itself did not reach statistical significance, it was almost identical to the significant among-individual effect of age. As such, we expect individuals to store increasing amounts of mercury in internal tissues between successive moulting events. Alternatively, however, older birds may improve their foraging efficiency or shift towards prey of higher trophic levels as they age (e.g. known for kleptoparasites in our colony (Garcia et al., 2020), but not for provisioning parents (Cansse et al., 2024)), thereby consistently ingesting, but also excreting, greater amounts of mercury. Such processes may vary across ages and feather types, potentially explaining why body and primary feathers exhibit clearer age effects than tail feathers. Either way, the observed age effect suggests that interpreting mercury concentrations in feathers of adult birds would ideally involve consideration of the age of the individuals that were sampled, at least when the sample sizes are limited, although evaluating the generality of the age effect represents an important direction for future research.

## Conclusion

Our study shows that although mercury concentrations in common tern feathers are positively correlated among feather types, their absolute levels depend on feather type and feather-type specifically on age. Mercury levels in body feathers were positively correlated with those in blood and therefore appear to reflect repeatable short-term dietary exposure to this pollutant, whereas primary and tail feathers integrate mercury over longer time periods and in response to internal redistribution. Given their strong correlation with blood mercury levels, their ability to pick up age effects, and the fact that they can be most little-invasively sampled from live birds, we advocate for the use of body feathers for biomonitoring purposes. However, future research should further explore the sources of mercury in feathers, for instance by combining stable isotope analyses with mercury measurements, and/or by linking mercury levels in internal organs with those in blood and feathers of the same individuals.

## Supplementary Information

Below is the link to the electronic supplementary material.

ESM 1 (200 KB DOCX)

## Data Availability

Data will be made available on request.
