# Supplementary material for: Differential mercury accumulation and links to blood mercury levels across feather types in a long-lived seabird
Source: Environ Monit Assess. 2026 Apr 15;198(5):460. doi: 10.1007/s10661-026-15310-4 (PMC13079512; doi:10.1007/s10661-026-15310-4)
Supplement: Supplementary file 1 — (200 KB DOCX) [file 10661_2026_15310_MOESM1_ESM.docx]

**Supplemental Material**

**to**

**Differential mercury accumulation and links to blood mercury levels across feather types in a long-lived seabird**

Justine Bertram^a,*^, Elias Garsi^a^, Coraline Bichet^b^, Nathalie Kürten^a^, Peter J. Schupp^c,d^, Sandra Bouwhuis^a^

^a^ Institute of Avian Research, An der Vogelwarte 21, 26386 Wilhelmshaven, Germany

^b^ Centre d’Etudes Biologiques de Chizé (CEBC), CNRS-La Rochelle Université, Villiers‑en‑Bois, France

^c^ Carl von Ossietzky Universität Oldenburg, Department for Chemistry and Biology of the Marine Environment, Terramare, 26382 Wilhelmshaven, Germany

^d^ Helmholtz Institute for Functional Marine Biodiversity at the University of Oldenburg
26129 Oldenburg, Germany


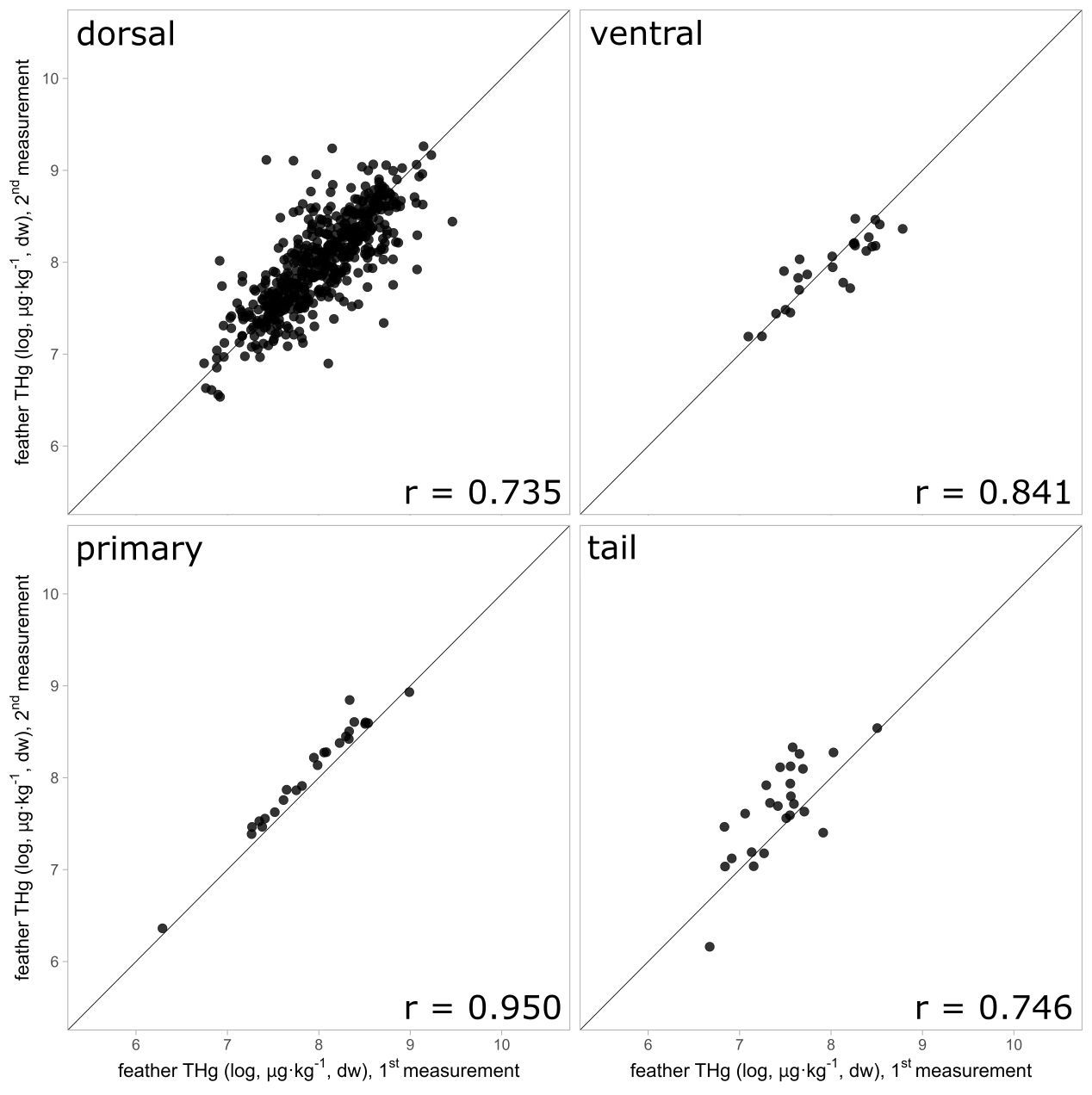


**Fig. S1**: Correlation (r) between sets of two total mercury concentration measurements of feathers of four types collected from the same common tern in the same year. Points represent the raw data, and the line represents the x = y line.
